# Biceps tenotomy versus tenodesis: patient-reported outcomes and satisfaction

**DOI:** 10.1186/s13018-020-1581-3

**Published:** 2020-02-18

**Authors:** Justin O. Aflatooni, Brett D. Meeks, Andrew W. Froehle, Kevin F. Bonner

**Affiliations:** 1 Eastern Virginia Medical School, 700 West Olney Ave, Norfolk, VA USA; 2 Department of Orthopaedic Surgery, Wright State University, Dayton, OH USA; 3 Jordan-Young Institute, Orthopaedic Surgery and Sports Medicine, Virginia Beach, VA USA

**Keywords:** Biceps tendon, Shoulder arthroscopy, Downsides, Spasms/cramping, Shoulder pain

## Abstract

**Background:**

Biceps tenotomy and tenodesis are surgical treatments for pathology of the proximal tendon of the long head of the biceps. There is debate over which procedure provides better patient outcomes.

**Purpose:**

Compare patient-reported outcomes and satisfaction between biceps tenotomy and tenodesis.

**Methods:**

This retrospective cohort study including all patients undergoing arthroscopic biceps tenodesis or tenotomy as part of more extensive shoulder surgery with a single surgeon. Concomitant procedures included rotator cuff repair, subacromial decompression, acromioclavicular joint resection, and debridement. Patients 36–81 years old were contacted by phone at > 2-year post-operatively to complete a biceps-specific outcome questionnaire. Subject decision not to participate was the sole exclusion criterion. Satisfaction scores and frequencies of potential biceps-related downsides (biceps cramping/spasms, biceps pain, shoulder pain, weakness, cosmetic deformity) were analyzed for the effects of procedure, sex, and age.

**Results:**

Satisfaction score distributions were similar between patients with tenodesis and patients with tenotomy (*χ*^2^ = 8.34, *P =* 0.08), although slightly more patients with tenodesis than patients with tenotomy reported being satisfied or very satisfied (96% versus 91%). Perceived downsides occurred more frequently among patients with tenotomy than in patients with tenodesis: 59% of patients with tenotomy reported ≥ 1 downside, versus 37% of patients with tenodesis (*P* < 0.01). In patients reporting ≥ 1 downside, distributions of total downsides differed between procedures (*χ*^2^ = 10.04, *P =* 0.04): patients with tenotomy were more likely to report multiple concurrent downsides than were patients with tenodesis (31% versus 16%). Each individual downside tended to be reported as present by a greater proportion of patients with tenotomy than patients with tenodesis. Sex had no effect on satisfaction or downsides, but there was a trend for older patients to report higher satisfaction and fewer downsides.

**Conclusions:**

Biceps tenotomy and tenodesis are both viable treatments for proximal biceps tendon pathology, yielding high patient satisfaction. There were trends toward greater satisfaction and fewer problems in patients with tenodesis. Still, younger patients with tenodesis did report perceived downsides. Alternatively, older patients tended to be more satisfied with both procedures overall. Regardless of procedure, most patients receiving either tenotomy or tenodesis would undergo their respective surgery again.

**Level of Evidence:**

Level III evidence, retrospective comparative cohort study

## Introduction

Pathology of the proximal long head of the biceps brachii tendon (LHBBT) can occur in isolation but is also commonly associated with other shoulder pathologies like impingement and rotator cuff tears [1]. The biceps tendon is therefore often addressed intraoperatively during concomitant surgery [1, 2]. Tenotomy and tenodesis are two accepted alternative procedures used to treat proximal biceps pathology and superior labrum anterior-posterior (SLAP) tears in the proper setting [2–8]. However, debate exists over which procedure provides better surgical outcomes, and studies have failed to demonstrate clear superiority of one procedure over the other [9].

Advantages of biceps tenotomy include being expeditious, simpler, and more cost-effective. Tenotomy also obviates some complications seen with biceps tenodesis, such as technical- and hardware-related problems, persistent shoulder pain, humeral fracture, neurovascular injury, complex regional pain syndrome, delayed failure, and other inherent surgical risks [10–12]. Drawbacks to biceps tenotomy include formation of a “popeye” sign, biceps muscle cramping and pain, shoulder pain, and biceps muscle weakness with certain activities. Boileau et al. reported that a popeye sign is present in 62% of patients with tenotomy [4]. Indeed, other studies found that a popeye sign was more common among patients with tenotomy than patients with tenodesis, as would be expected [5, 13]. However, despite this increase in cosmetic deformity among patients with tenotomy, the deformity is not always noticed by the patient, and the cosmetic differences between tenotomy and patients with tenodesis are not always significant nor of perceived consequence to the patient [2, 4, 13, 14]. Nord et al. found that biceps shape was preserved in 90% of patients with tenotomy [15]. A small randomized prospective study similarly found no difference between tenotomy and tenodesis procedures for popeye sign or strength differences [16]. Overall, complication rates for tenodesis and tenotomy are relatively low, with most patients reporting positive outcomes [17–19].

Since evidence-based indications for the use of one method over the other remain unclear, the choice is usually guided by surgeon preference, experience, and individual patient factors. This study focuses on the latter aspect, analyzing patient-centered outcomes of biceps tenotomy and tenodesis performed by a single surgeon. The study’s goal was to compare each procedure for patient-reported satisfaction and potential biceps-specific downsides and to analyze the effects of age and sex on these outcomes. We hypothesized that tenotomy would have higher rates of popeye sign but that overall patient satisfaction would be similar between procedures, with no significant effects of sex or age on the respective outcomes.

## Methods

This study is a continuation of the previously published “Patient Satisfaction After Biceps Tenotomy” by Meeks et al. [18] All procedures were performed by a single surgeon, concomitantly with subacromial decompression, acromioclavicular joint resection, and/or debridement surgeries for other shoulder pathologies (Table 1). Patients underwent tenotomy or tenodesis for proximal biceps or superior labral complex pathology, with ultimate treatment based on patient factors and surgeon decision. For the tenotomy procedure, the biceps tendon was released with electrocautery at the insertion into the superior labrum. All tenodesis procedures were performed arthroscopically at the distal border of the bicipital groove. The biceps tendons were released in the same method as those undergoing tenotomy. The tendons were then brought out through a portal directly anterior to the inferior bicipital groove and shortened by approximately 2.5–3 cm in order to optimize the length-tension relationship of the biceps. A non-absorbable interlocking stitch was placed into the end of the tendon and used to insert the tendon into a unicortical 23-mm tunnel/socket within the tenodesis device. Finally, the tendon was fixated with an interference screw (Arthrex Tenodesis screw, Naples, FL). Patients with tenotomy tended to be treated earlier in the study period (2009–2011), and their results were previously reported in Meeks et al. [18]. The same data collection methods were used for patients with tenodesis later in the study period (2013–2015) to allow direct comparison of cohorts [18].

**Table 1:** Patient demographics and concomitant procedures

Demographics	Tenodesis	Tenotomy
Participants	111	104
Male (%)	80 (72%)	48 (46%)
Female (%)	31 (28%)	56 (54%)
Mean age ± SD	58.9 ± 8.8	63.5 ± 8.6
L (R) shoulder	35 (76)	33 (71)
Follow-up time, months, mean (range)	30.7 (22–43)	38.4 (22–57)
**Procedures performed concurrently,*N*(%)**		
RCR with or without SAD	56 (51)	64 (61)
AC joint resection	30 (27)	8 (8)
SAD	18 (16)	30 (29)
Debridement of tear/joint ± RCR	7 (6)	2 (2)

Tenodesis patient demographic information along with concomitant procedures performed in the tenotomy and tenodesis cohorts, and the number of patients that received each. There were significantly more female tenotomy patients (*P* < 0.01) and the mean age was higher relative to tenodesis patients (*P* < 0.01)

*SD* standard deviation, *RCR* rotator cuff repair, *SAD* subacromial decompression, *AC* acromioclavicular

The local Institutional Review Board (IRB) approved all study procedures prior to commencement. Data collection included retrospective chart review and a phone interview, during which verbal consent was obtained and a biceps-specific survey (Appendix) was administered. All answers, including whether a popeye deformity developed, were subjectively assessed by the patient themselves and, therefore, specifics (i.e., for biceps pain, delineation between groove versus muscle pain) were not distinguished. The survey, which has previously been published, focused on specific questions related to management of the biceps tendon [18].

Of 149 patients with tenodesis contacted, 111 consented to participate (75% response rate), 32 were lost to follow-up, and 6 declined to participate. Mean age was 58.9 ± 8.8 years (range 36–81 years), with a sex distribution of 72% male/28% female (Table 1). Mean follow-up time was 30.7 ± 6.0 months (range 22–43 months). Of 123 eligible patients with tenotomy, 104 were enrolled (85% response rate), with 17 patients lost to follow-up, and 2 declining to participate. Mean age was 63.5 ± 8.6 years (range 40–81 years), and the cohort was 46% male/54% female. Mean follow-up time was 38.4 ± 5.8 months (range 22–57 months) (Table 1) [18].

Statistical analysis was performed using SAS 9.4 (Cary, NC), with significance set to *α* = 0.05. Initial analysis focused on describing the tenodesis sample, mirroring previous work with the tenotomy sample [18], and testing for effects of age and sex on tenodesis-specific outcomes. To analyze the effects of age on outcomes, Kruskal-Wallis tests were used to compare age distributions of respondents at each satisfaction level. Wilcoxon-Mann-Whitney tests were then used to compare average ages between “yes” and “no” respondents for each type of downside of the procedure. To test for the effects of sex on outcomes, a chi-square test was used to compare the distributions of satisfaction scores in males and females. Fisher’s exact tests were used to compare the sex distributions of “yes” and “no” respondents for each type of downside.

The second set of analyses compared the tenodesis group to the tenotomy cohort. This was a convenience sample, and thus, no a priori power analysis was performed before collecting data. An independent samples *t* test was used to test for an age difference between the two groups, and a Fisher’s exact test was used to compare the groups’ sex distributions, to determine how demographically similar or dissimilar the cohorts were. A chi-square test was used to compare satisfaction score distributions between the groups. Fisher’s exact tests were used to analyze the frequencies of reported downsides in each surgical procedure group, and a chi-square test was used to compare the procedures in terms of the distributions of total number of downsides per individual patient. Odds ratios were also calculated for each reported downside, comparing patients with tenotomy to patients with tenodesis.

To investigate which factors contributed most to postsurgical satisfaction scores and reported downsides, we conducted a series of exploratory multiple ordinal logistic regression analyses. First, we wished to determine the effects of age, sex, procedure, and the number of downsides on satisfaction scores as the outcome of interest. We initially treated the total number of concurrent downsides per patient as an ordinal variable. On the basis of preliminary results, we then generated a final model treating downsides as a categorical variable with two levels: 0 = 0 downsides and 1 = ≥1 downside. We also conducted a secondary set of ordinal logistic regression analyses to determine the effects of age, sex, and procedure on the presence of downsides. In addition to overall model and factor-specific regression parameters, we also calculated odds ratios for each factor. Each set of analyses was conducted using a stepwise process, first generating a preliminary model including all factors, then removing non-influential factors to build a final model.

## Results

### Tenodesis cohort

**Fig. 1:**
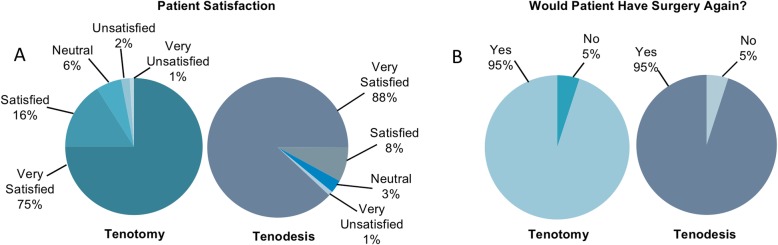
Patient-reported satisfaction after biceps tenotomy or tenodesis and concomitant procedure. The first pie chart is for tenotomy, followed by the tenodesis chart. **a** Percentage of patients reporting satisfaction. Among patients with tenodesis, ninety-eight, nine, three, and one gave a score of 5 (“very satisfied”), 4 (“satisfied”), 3 (“neutral”), and 1 (“very unsatisfied”), respectively, with no patients giving a score of 2 (“unsatisfied”). Among patient with tenotomy, seventy-eight, seventeen, six, two, and one gave a satisfaction score of 5, 4, 3, 2, and 1, respectively. **b** Percentage of patients reporting they would have the same surgery again. Among patients with tenodesis, 104 reported they would have the procedure again. Among patients with tenotomy, 91 reported that they would have the procedure again

As seen in Fig. 1, a majority of the patients that received tenodesis reported both that they were satisfied with their outcome and that they would have their procedure again. There was a trend toward older age among respondents with higher satisfaction scores: median ages within each satisfaction level were 60 (5), 56 (4), 51 (3), and 52 (1) (Kruskal-Wallis: *P* = 0.15) years old, respectively. With regard to satisfaction score distributions by sex, 97% of males and 96% of females were either satisfied or very satisfied (*χ*^2^ = 5.06, *P =* 0.17).

**Fig. 2:**
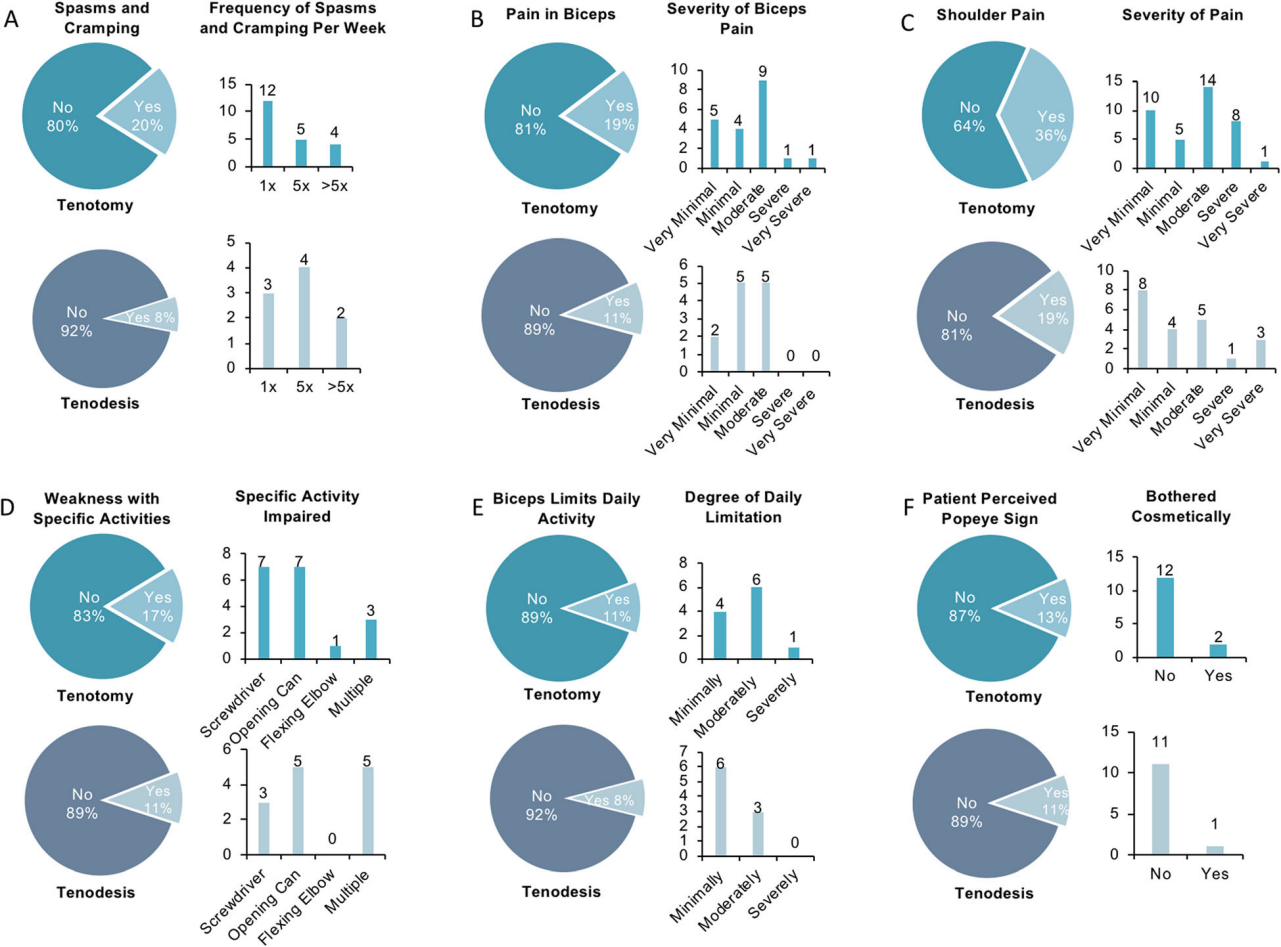
Pie charts comparing patient-reported downsides following either biceps tenotomy or tenodesis, and an associated bar graph providing further details about that downside. The top pie chart, for each downside, is for tenotomy followed by the lower chart for tenodesis. **a** Patients reporting biceps spasms and cramping with a graph detailing the frequency of this downside. **b** Patients reporting biceps muscle pain with a graph rating their pain 1–5. **c** Patients reporting shoulder pain with a graph rating their pain 1–5. **d** Patients reporting activity-specific weakness with an accompanying graph detailing which activity patients noticed weakness. **e** Patients reporting daily activity limitation with a graph rating the level of limitation. **f** Patients noticing a popeye sign with a graph detailing how many were cosmetically bothered by this

Downsides were relatively uncommon among patients with tenodesis, 70 of whom (63%) reported having none. Still, 41 patients (37%) reported having one or more downsides (1, 23 patients; 2, 7 patients; 3, 7 patients; 4, 3 patients; 5, 1 patient). Nine patients (8%) reported biceps spasms/cramping, 12 patients (11%) reported biceps pain, 21 patients (19%) reported shoulder pain, 12 patients (11%) reported weakness in biceps-related activity, and 9 patients (8%) reported limitations in daily activities due to biceps-reported downsides. Finally, the popeye sign occurred in 12 patients (11%), only one of whom reported being cosmetically bothered (Fig. 2). This was primarily due to a presumed delayed rupture or failure of the tenodesis, which likely occurred in the first 2 months post-operatively. However, no patients wished to undergo revision tenodesis.

Rates of each downside were broadly similar between sexes as follows: spasms/cramping (male 8%, female 10%, *P* = 0.71), biceps pain (male 9%, female 16%, *P* = 0.31), shoulder pain (male 18%, female 23%, *P* = 0.59), weakness (male 11%, female 10%, *P* = 0.81), limitations (male 8%, female 10%, *P* = 0.71), and popeye sign (male 10%; female 13%, *P* = 0.74). For each downside, patients reporting their presence tended to be younger than those for whom downsides were not reported (reported values are mean ± SD): spasms/cramping (present, 51.4 ± 4.6 years; absent, 59.6 ± 8.8 years; *P* < 0.01), biceps pain (present 54.7 ± 9.3 years, absent 59.4 ± 8.7 years, *P* = 0.10), shoulder pain (present 56.2 ± 10.2 years, absent 59.5 ± 8.4 years, *P* = 0.25), weakness (present 52.0 ± 6.7 years, absent 59.7 ± 8.7 years, *P* < 0.01), limitations (present 47.0 ± 4.8 years, absent 60.0 ± 8.3 years, *P* < 0.01), and popeye sign (present 56.5 ± 8.5 years, absent 59.2 ± 8.9 years, *P* = 0.30).

### Tenotomy cohort

Among patients with tenotomy, a majority reported that they are both satisfied with their outcome and that they would have their procedure again (Fig. 1). With the exception of patients giving a satisfaction score of 2, median age was relatively consistent across satisfaction score groups: 64 (5), 62 (4), 62 (3), 70 (2), and 60 (1) (Kruskal-Wallis: *P* = 0.40) years, respectively. With regard to sex, 96% of women were satisfied or very satisfied, compared with 87% of men, and whereas no women gave scores of 1 or 2, 5% of men did (*χ*^2^ = 8.94, *P =* 0.06).

Twenty-one patients (20%) reported biceps spasms/cramping, 20 patients (19%) reported biceps pain, 37 patients (36%) reported shoulder pain, 18 patients (17%) reported weakness in biceps-related activity, and 11 patients (11%) reported limitations in daily activities due to their biceps-reported downsides. Finally, the popeye sign occurred in 14 patients (13%), only two of whom reported being cosmetically bothered by it (Fig. 2) [18].

Rates of each reported downside tended to be at least slightly higher in men than in women, as follows: spasms/cramping (male, 25%; female, 14%; *P* = 0.22), biceps pain (male, 24%; female, 14%; *P* = 0.32), shoulder pain (male, 40%; female,31%; *P* = 0.41), weakness (male, 18%; female, 16%; *P* = 0.80), limitations (male, 16%; female, 4%; *P* = 0.04), and popeye sign (male, 18%; female, 10%; *P* = 0.28). With the exceptions of spasms/cramping and popeye sign, mean ages were similar between patients reporting the presence versus absence of each downside (reported values are mean ± SD): spasms/cramping (present, 61.7 ± 8.7 years; absent, 64.0 ± 8.6 years; *P* = 0.25), biceps pain (present, 62.8 ± 8.6 years; absent, 63.7 ± 8.7 years; *P* = 0.63), shoulder pain (present, 63.9 ± 9.0 years; absent, 63.3 ± 8.5 years; *P* = 0.63), weakness (present, 63.7 ± 9.9 years; absent, 63.5 ± 8.4 years; *P* = 0.78), limitations (present, 63.0 ± 11.2 years; absent, 63.6 ± 8.4 years; *P* = 0.59), and popeye sign (present, 60.3 ± 7.8 years; absent, 64.1 ± 8.7 years; *P* = 0.10).

### Comparison of tenodesis and tenotomy cohort outcomes

Compared with the tenodesis cohort, the tenotomy cohort was significantly older and included a higher proportion of females. Table 1 displays these data along with the type of procedure and number of patients that received a concurrent operation in each cohort. More patients with tenotomy received either concomitant acromioclavicular joint resection (*P* < 0.01) or isolated subacromial decompression without rotator cuff repair (RCR) (*P* = 0.03).

**Fig. 3:**
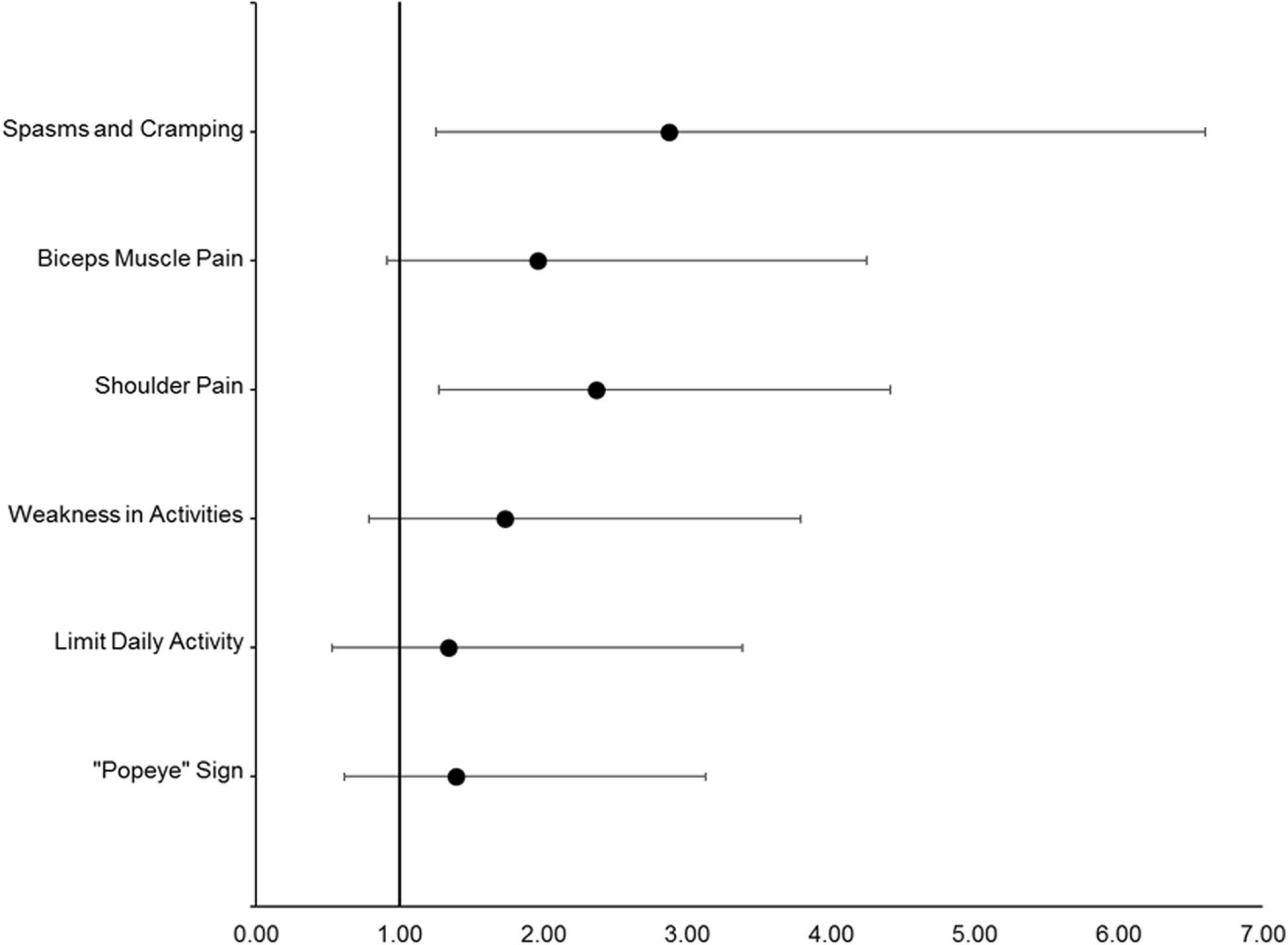
Forest plot of odds ratios of the likelihood of patients with tenotomy to experience a negative outcome relative to patients with tenodesis. Spasms/cramping (tenotomy 20%; tenodesis 8%; *P* = 0.02; OR = 2.87, 95% CI 1.25–6.60); biceps pain (tenotomy 20%; tenodesis 11%; *P* = 0.09; OR = 1.96, 95% CI 0.91–4.25); shoulder pain (tenotomy 36%; tenodesis 19%; *P* = 0.01; OR = 2.37, 95% CI 1.27–4.41); weakness (tenotomy 17%; tenodesis 11%; *P* = 0.24; OR = 1.73, 95% CI 0.79–3.79); limitations (tenotomy 11%; tenodesis 8%; *P* = 0.64; OR = 1.34, 95% CI 0.53–3.38); and the popeye sign (tenotomy 14%; tenodesis 11%; *P* = 0.54; OR = 1.39, 95% CI 0.62–3.13)

Overall distributions of satisfaction scores were similar between the two cohorts, with very few reports of dissatisfaction (*χ*^2^ = 8.34, *P =* 0.08), although more patients with tenodesis than patients with tenotomy reported being satisfied or very satisfied (96% versus 91%) with their respective procedures. Perceived downsides occurred more frequently among patients with tenotomy than in patients with tenodesis: 59% of patients with tenotomy reported at least one downside, versus 37% of patients with tenodesis (*P* < 0.01). In patients reporting at least one downside, distributions of total number of downsides differed between procedures (*χ*^2^ = 10.04, *P =* 0.04), such that patients with tenotomy were more likely to report multiple concurrent downsides than were patients with tenodesis (31% versus 16%). Additionally, each downside tended to be reported by a greater proportion of patients with tenotomy than patients with tenodesis, but only shoulder pain and biceps brachii muscle spasms and cramping were shown to be significantly different between the two populations (see Fig. 3). Relative risk analysis showed that patients with tenotomy were more than twice as likely to experience spasms/cramping, and almost twice as likely to experience shoulder pain than patients with tenodesis (Table 2).

**Table 2:** Relative risk of downsides

Reported downside	RR	95% CI	Significance
Spasms/cramping	2.49	1.20–5.19	Significant; CI does not include 1
Biceps pain	1.78	0.92–3.45	Not significant; CI includes 1
Shoulder pain	1.88	1.18–2.99	Significant; CI does not include 1
Weakness	1.60	0.81–3.16	Not significant; CI includes 1
Limits daily activities	1.30	0.56–3.02	Not Significant; CI includes 1
Popeye Sign	1.33	0.66–2.71	Not Significant; CI includes 1

Relative risk for “Yes” responses to downsides in tenotomy (exposure) versus tenodesis (non-exposure)

### Factors contributing to satisfaction scores and downsides

The preliminary ordinal logistic model determining predictors of satisfaction score was statistically significant (*P* < 0.01) but with no significant effects of age (*P* = 0.57; OR = 1.01, 95% CI = 0.97–1.07), sex (*P* = 0.28; OR = 0.616, 95% CI = 0.26–1.48), or procedure (*P* = 0.15; OR = 1.96, 95% CI = 0.78–4.91). The only significant predictor in this model was the total number of downsides (overall effect *P* < 0.01), where a greater number of downsides was related to higher likelihood of a lower satisfaction score. Comparing cumulative downside levels, however, the only significant increase in risk for lower satisfaction occurred when comparing patients with one downside to those with none (*P* = 0.01; OR = 0.194, 95% CI = 0.056–0.673). Remaining increases in the total number of downsides (e.g., 2 versus 1, 3 versus 2, etc.) had no significant effects on satisfaction scores (for each, *P* ≥ 0.12; all OR 95% CIs included 1.00).

Since the only significant effect of increasing total downsides occurred at the step from zero to one, we performed a second ordinal logistic analysis of factors predicting satisfaction score, treating downsides as a binary variable (0: no downside, 1: ≥ 1 downside). This initial model was statistically significant (*P* < 0.01), with significant effects of procedure (*P* = 0.05; OR = 2.40, 95% CI = 1.02–5.65) and downside (*P* < 0.01; OR = 0.08, 95% CI = 0.03–0.24). The effect of age-approached significance (*P* = 0.06), and the lower bound of the 95% CI of the OR was 1.00 (OR = 1.04, 95% CI = 1.00–1.09), so we kept age in the final model. Sex was removed, since we interpreted its effect as minimal (*P* = 0.17; OR = 0.56, 95% CI = 0.25–1.27). The final model effects were as follows: procedure (*P* = 0.05; OR = 2.33, 95% CI = 1.00–5.45), downsides (*P* < 0.01; OR = 0.08, 95% CI = 0.03–0.24), and age (*P* = 0.04; OR = 1.05, 95% CI = 1.00–1.09). Therefore, patients with tenodesis, patients without downsides, and older patients all tended to have higher satisfaction scores (Table 3).

**Table 3:**
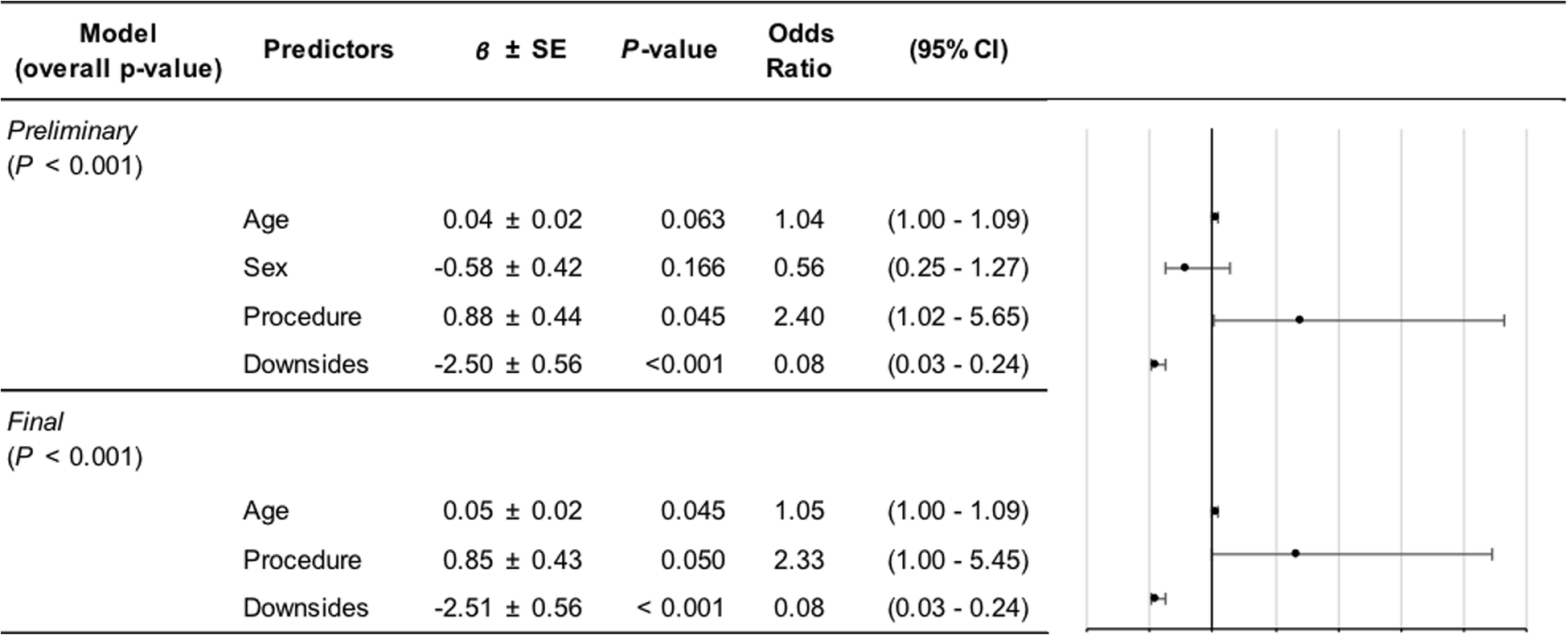
Ordinal logistic regression results for predictors of satisfaction scores

To determine predictors of the presence of any postsurgical downsides, a statistically significant overall preliminary model was found (*P* < 0.01) with factor effects as follows: procedure (*P* < 0.01; OR = 0.35, 95% CI = 0.20–0.63); age (*P* = 0.11; OR = 0.97, 95% CI = 0.94–1.01); and sex (*P* = 0.63; OR = 1.16, 95% CI = 0.64–2.08). We also analyzed total downsides per patient as an ordinal outcome variable, finding a significant overall model (*P* < 0.01) in which procedure (*P* < 0.01; OR = 0.34, 95% CI = 0.19–0.59) and age (*P* = 0.01; OR = 0.96, 95% CI = 0.93–0.99) had significant effects, but sex did not (*P* = 0.47; OR = 1.22, 95% CI = 0.71–2.11). Odds ratios and their 95% confidence intervals for procedure and age did not change substantively when sex was removed from either model. Patients with tenodesis and older patients tended to report fewer postsurgical downsides (Table 4).

**Table 4:**
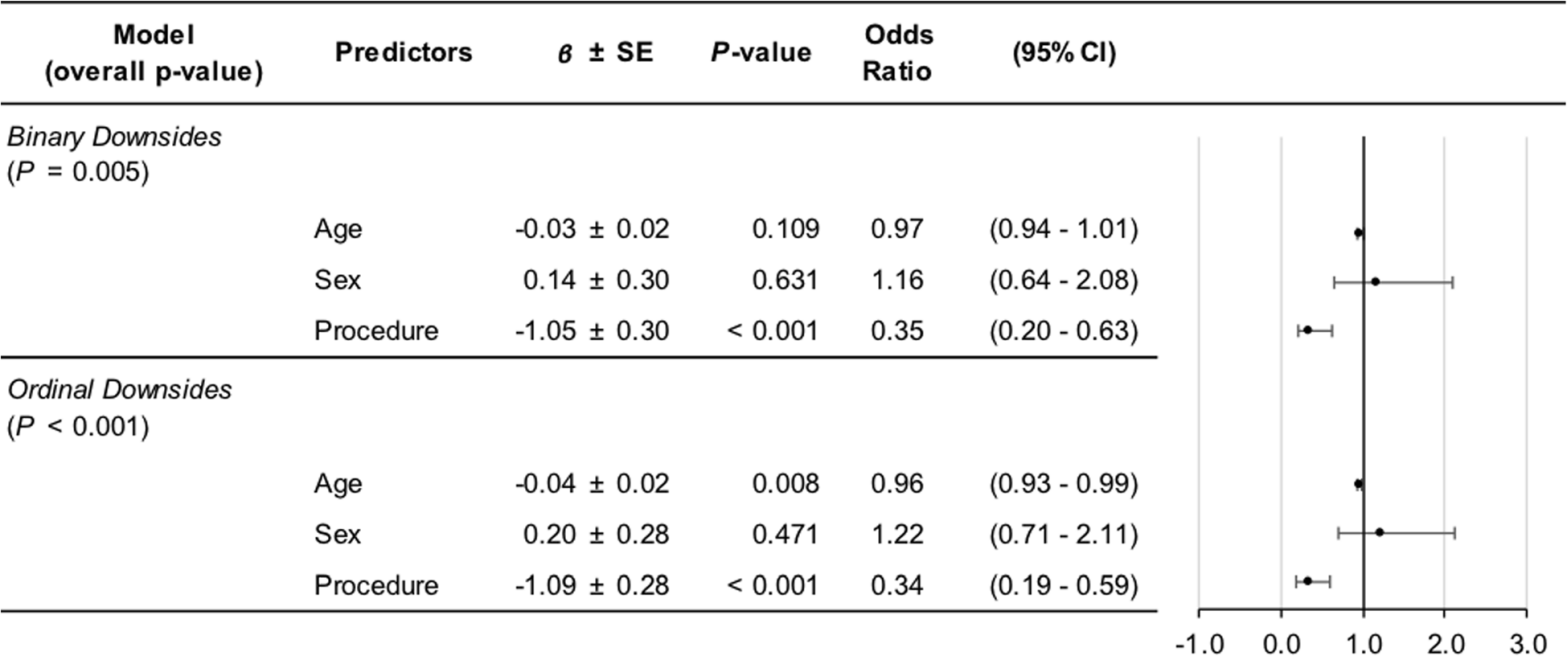
Ordinal logistic regression results for predictors of downsides

## Discussion

Our results show that very high patient satisfaction can be achieved with either tenotomy or tenodesis to address proximal biceps pathology. Recent literature is consistent with this finding, as many studies have found no significant differences in pain, function, or limitations between tenotomy and patients with tenodesis [6, 16, 20–24]. However, others have shown increased shoulder pain and loss of supination power with biceps tenotomy, with many authors favoring biceps tenodesis in younger patients with a higher work and activity level [8, 20, 23]. Although we found broad satisfaction among both tenotomy and patients with tenodesis, a trend toward higher satisfaction was associated with tenodesis.

In terms of downsides, although a marginally higher proportion of patients with tenotomy reported limitations and the popeye sign (+ 3%) compared with patients with tenodesis, those disparities were larger for weakness (+ 6% in tenotomy) and even greater for spasms/cramping (+ 12%), biceps pain (+ 9%), and shoulder pain (+ 17%). Previous studies had similar results. Castricini et al. and Hassan et al. found little to no significant differences in downsides between the two procedures, except that patients with tenotomy experienced more shoulder pain as well as biceps spasms and cramping [19, 25]. Lee et al. also found no difference in outcomes of function or pain between tenodesis and patients with tenotomy [23].

It is worth noting that many subjects in both of our groups underwent other procedures (Table 1). Two patients with tenotomy had irreparable rotator cuff tears, which were not seen in our tenodesis group. Clearly, it is impossible to discern if patient satisfaction is primarily related to concomitant procedures. In many cases, RCR was the major component of the procedure which is why we developed a questionnaire specific to biceps-related problems. A systematic review and meta-analysis comparing tenotomy and tenodesis procedures performed concomitantly with RCR found that patients undergoing RCR with tenotomy were significantly more likely to generate a lower Constant-Murley score and develop a popeye deformity [21, 22]. However, these differences were not clinically significant, and there was no significant difference in patient satisfaction [21]. Another meta-analysis found similar results but found significantly more biceps cramping and popeye deformity in patients with tenotomy [7]. With the exception of the popeye deformity findings, all of these findings are consistent with our results.

When looking at factors impacting satisfaction, our results suggest that older patients, patients with tenodesis, and patients with fewer downsides tend to have higher satisfaction scores. These factors appear to be interrelated, in that fewer downsides tended to be reported with tenodesis in general (despite the tenodesis cohort being younger than the tenotomy cohort), and older patients tended to report fewer downsides. The relationship between age and downsides may be a product of baseline function: older patients may have less function or more pain to begin with, and so postsurgical gains may be seen as a benefit. In contrast, younger patients who are less limited to begin with may expect a greater return of function following surgery, and may thus have a greater tendency to report downsides when their expectations are not met, with an impact on overall satisfaction.

Some of those effects may be procedure-specific. Within tenodesis, older patients tended to be more satisfied and were less likely to report downsides. The sexes tended to be similarly satisfied with their tenodesis results, and males and females had similar outcomes in terms of downsides, although there was a trend toward higher rates of biceps and shoulder pain in females. In contrast, with tenotomy there appeared to be very little effect of age on either satisfaction or downsides, but women reported fewer downsides and were 10% more satisfied than men. These findings stand in contrast to Galdi et al. who found that when preoperatively learning about each procedure, females tended to prefer tenodesis citing worry over a popeye deformity [26]. Alternatively, men favored tenotomy citing the shorter recovery time, lack of hardware, and decreased incidence of bicipital groove pain as motivating factors [26]. Other studies have found that a popeye deformity was more likely to both develop and become severe in men versus women who received tenotomy [6]. Despite these results, we found only a 3% difference in reported popeye deformity between our study groups.

Delayed failure of tenodesis fixation may help explain the similar frequency of a popeye sign between our groups. We found a delayed rupture or failure rate of 11% in our tenodesis group, consistent with other studies reporting delayed failure or rupture rates up to 20% [27]. This failure/rupture of the tenodesis is responsible for the incidence of popeye sign associated with tenodesis. Theoretically, if no failures occurred there would likely be a more significant difference in popeye deformity and perhaps other downsides as well. The senior author has found that delayed failures do occur with interference screw fixation devices, and is most common within 3–12 weeks post-op. This is consistent with other authors’ findings [27]. We have found that this can occur even when healthy tendons undergo tenodesis.

## Limitations

In this study, every patient received a biceps tenodesis or tenotomy in the context of concomitant procedures for coexisting shoulder pathology. These concurrent procedures may have influenced patient responses to the questionnaire. For example, many patients underwent concomitant rotator cuff repair, which may have contributed to perceived downsides or improvement that was attributed to the respective biceps procedure. Furthermore, the biceps-specific questionnaire used in this study has not been validated by external sources or other studies and may contribute to the limitations of this study. Additionally, patients were not randomized but rather received biceps tenodesis or tenotomy after consulting with the senior author, which may have influenced our results due to selection bias. This also resulted in a heterogeneous distribution between the groups with patient age being significantly different between the two. A repeat MRI to determine successful healing of the tenodesis was not performed. Popeye deformity in the tenodesis group was typically consistent with the patient feeling a “pop” within 2 months following surgery though failure was not confirmed via physician exam. Confirmatory MRI was also not performed in most cases since none of the patients desired revision tenodesis, and thus, this would not have altered the patient’s care.

## Conclusions

The results show that biceps tenotomy and tenodesis are both viable treatments for proximal biceps tendon pathology, yielding high patient satisfaction in the context of concomitant shoulder surgery. There were trends toward greater satisfaction and fewer problems in patients with tenodesis. Still, younger patients with tenodesis did report perceived downsides. Patients in both groups reported with equal frequency (95%) that they would repeat the procedure.

## Data Availability

The original datasets generated and analyzed during the current study are not publicly available due to protection of private health information. However, a de-identified dataset may be provided by the corresponding author on reasonable request.

## Appendix

### Form 1

Questionnaire used to assess patient satisfaction among arthroscopic biceps patients with tenotomy (Meeks et al.) or tenodesis procedure.

## Abbreviations

CI: 95% confidence interval
IRB: Institutional review board
LHBBT: Long head biceps brachii tendon
MRI: Magnetic resonance imaging
OR: Odds ratio
RCR: Rotator cuff repair
SD: Standard deviation
SLAP: Superior labrum anterior-posterior
